# Disparities in pediatric drug-resistant epilepsy care

**DOI:** 10.1007/s00381-023-05854-y

**Published:** 2023-02-17

**Authors:** Melissa A. LoPresti, Lu Zhang, Sandi Lam

**Affiliations:** Division of Pediatric Neurosurgery, Department of Neurosurgery, Northwestern University Feinberg School of Medicine, Lurie Children’s Hospital, 225 E Chicago Ave, Box 28, Chicago, IL 60611 USA

**Keywords:** Epilepsy, Surgical epilepsy, Disparities, Pediatric

## Abstract

**Introduction:**

Epilepsy affects millions of children worldwide, with 20–40% experiencing drug-resistant epilepsy (DRE) who are recommended for epilepsy surgery evaluation and may benefit from surgical management. However, many patients live with DRE for multiple years prior to surgical epilepsy referral or treatment or are never referred at all.

**Objective:**

We aimed to describe factors associated with referral for epilepsy surgery in the USA, in order to identify disparities in DRE, characterize why they may exist, and recognize areas for improvement.

**Methods:**

Pediatric patients diagnosed with DRE between January 1, 2004 and December 31, 2020 were identified from the Pediatric Health Information System (PHIS) Database. Patients treated with antiseizure medications (ASMs) only, ASMs plus vagus nerve stimulation (VNS), and ASMs plus cranial epilepsy surgery were studied regarding access to epilepsy surgery and disparities in care. This study used chi-square tests to determine associations between treatment time and preoperative factors. Preoperative factors studied included epilepsy treatment type, age, sex, race/ethnicity, insurance type, geographic region, patient type, epilepsy type, and presence of pediatric complex chronic conditions (PCCCs).

**Results:**

A total of 18,292 patients were identified; 10,240 treated with ASMs, 5019 treated with ASMs + VNS, and 3033 treated with ASMs + cranial epilepsy surgery. Sex was not found to significantly vary among groups. There was significant variation in age, census region, race/ethnicity, patient type, presence of PCCCs, diagnosis, and insurance (*p* < 0.001). Those treated surgically, either with VNS or cranial epilepsy surgery, were 2 years older than those medically treated. Additionally, those medically treated were less likely to be living in the Midwest (25.46%), identified as non-Hispanic white (51.78%), have a focal/partial epilepsy diagnosis (8.74%), and be privately insured (35.82%).

**Conclusions:**

We studied a large administrative US database examining variables associated with surgical epilepsy evaluation and management. We found significant variation in treatment associated with age, US census region, race/ethnicity, patient type, presence of PCCCs, diagnosis, and health insurance type. We believe that these disparities in care are related to access and social determinants of health, and we encourage focused outreach strategies to mitigate these disparities to broaden access and improve outcomes in children in the USA with DRE.

**Supplementary Information:**

The online version contains supplementary material available at 10.1007/s00381-023-05854-y.

## Introduction

Epilepsy affects millions of children worldwide, with 20–40% experiencing drug-resistant epilepsy (DRE) who are recommended for surgical evaluation and management [1, 2]. Surgical management can afford improved seizure control and severity, while reducing costs, healthcare utilization, and negative quality-of-life impacts long-term [3]. Despite this, many patients live with DRE for years prior to surgical epilepsy referral or treatment or are never referred at all.

Disparities in epilepsy care exist. In the USA, for example, the incidence of epilepsy is higher in Hispanic and African American populations, yet these groups are less likely to undergo epilepsy surgery and more likely to present in the emergency department with seizures [4]. A multitude of factors may contribute to this; however, social determinants of health are implicated. Known determinants including socioeconomic status, race/ethnicity, age, and gender, as well as other social/cultural, behavioral, biological, psychological, and environmental factors which influence the patient and environment, contributing to complex relationships in engagement and participation in care, thus contributing to disparities in care and outcomes [5]. While these may vary by city, state, region, and country, identifying how these impact patient outcomes is key to overcoming obstacles.

Understanding the factors which contribute to these disparities is key to reconciliation. Acknowledging how access barriers, communication obstacles, educational attainment and health literacy, patient-physician trust, fear of treatment, and social support contribute to disparities in care experienced by groups with adverse social determinants of health, we can begin to close the gap in care in the USA [4, 6]. Thus, we aimed to characterize factors associated with referral for epilepsy surgery nationwide in order to identify disparities in DRE and recognize areas for improvement in the USA.

## Methods

### Data

Pediatric patients diagnosed with DRE between January 1, 2004 and December 31, 2020 were identified from the Children’s Hospital Association’s (Lenexa, KS) Pediatric Health Information System (PHIS), a US administrative database that contains inpatient, emergency department, ambulatory, and observation encounter level data from more than 44 children’s hospitals in the USA. Longitudinal study was conducted after patients were assigned an identifier for subsequent encounters in the database. As this involved non-human subjects research, it was completed with Institutional Review Board exempt status.

### Study cohort and design

The study cohort was formed via retrospective query of the PHIS database using *International Classification of Diseases, 9th Revision, Clinical Modification* (ICD-9-CM) and *10th Revision, Clinical Modification* (ICD-10-CM) codes. Data was extracted for pediatric patients (ages 0 to 17 years) discharged between January 1, 2004 and December 31, 2020 with diagnosis codes of epilepsy (ICD-9-CM code 345.XX and ICD-10-CM code G40.XXX) or seizure (ICD-9-CM code 780.3X and ICD-10-CM code R56.X or R56.XX). These patients were then included if they met previously published algorithms reported for identifying epilepsy [7–11], including (1) at least 2 encounters with diagnosis code 345.XX or G40.XXX on separate dates in any visit; (2) at least 1 encounter with diagnosis code 345.XX or G40.XXX and at least 1 separate encounter on a different date with diagnosis code 780.3X or R56.X or R56.XX; (3) a primary diagnosis code 345.XX or G40.XXX and a therapeutic category code indicating antiepileptic medication; (4) at least 2 encounters with diagnosis code 780.3X or R56.X or R56.XX and code(s) for antiepileptic medication; or (5) an inpatient or emergency department visit with a primary diagnosis code 345.XX or G40.XXX. Patients with DRE from the above cohort were then selected using the diagnosis codes listed in Supplementary Table 1. Finally, we excluded patients without one full year of baseline data prior to the index date and patients with missing values on key variables.

Patients were then sorted into cohorts based on treatment with at least three antiseizure medications (ASMs) only, ASMs plus vagus nerve stimulation (VNS), and ASMs plus cranial epilepsy surgery. These 3 groups were studied regarding treatment type received. Those undergoing VNS and cranial epilepsy surgery were excluded from subgroup analysis as we aimed to look at access to surgical care and the cohort was defined in terms of first surgery. We thus excluded patients with multiple modalities of epilepsy surgery given the aim of the study design.

### Variables

**Fig. 1:**
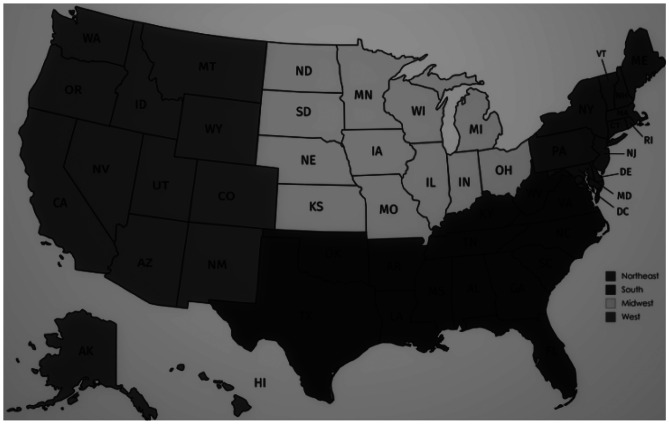
US census regions

Variables studied included epilepsy treatment type, age, sex, race/ethnicity, insurance type, geographic region, patient type, epilepsy type, and presence of pediatric complex chronic conditions (PCCCs). These were defined as epilepsy treatment type (medical treatment, VNS and cranial epilepsy surgery); age at index date (< 4, 4 to 11, and 12 to 17 years); gender (female, male); race/ethnicity (non-Hispanic white, non-Hispanic black, Hispanic, and others); insurance (Medicaid, private, and others); geographic region (Midwest, Northeast, South, and West) (Fig. 1); patient type at index date (inpatient, outpatient); epilepsy diagnosis by coding (focal/partial epilepsy, generalized epilepsy, and others); and presence of any pediatric complex chronic condition (CCCs).

The outcome studied was treatment type, including antiseizure medications (ASMs) only, ASMs plus vagus nerve stimulation (VNS), and ASMs plus cranial epilepsy surgery. Associations with preoperative factors and treatment time were examined.

### Statistical analysis

Frequencies and proportions of all factors were reported for the whole sample and each individual treatment group. Pearson’s chi-square tests were used to examine the associations between factors and treatment type. Analysis was done by using Statistical Analysis Software SAS^®^ 9.4 (SAS Institute, Cary, NC). The significance level was set at *p* < 0.05.

## Results

**Fig. 2:**
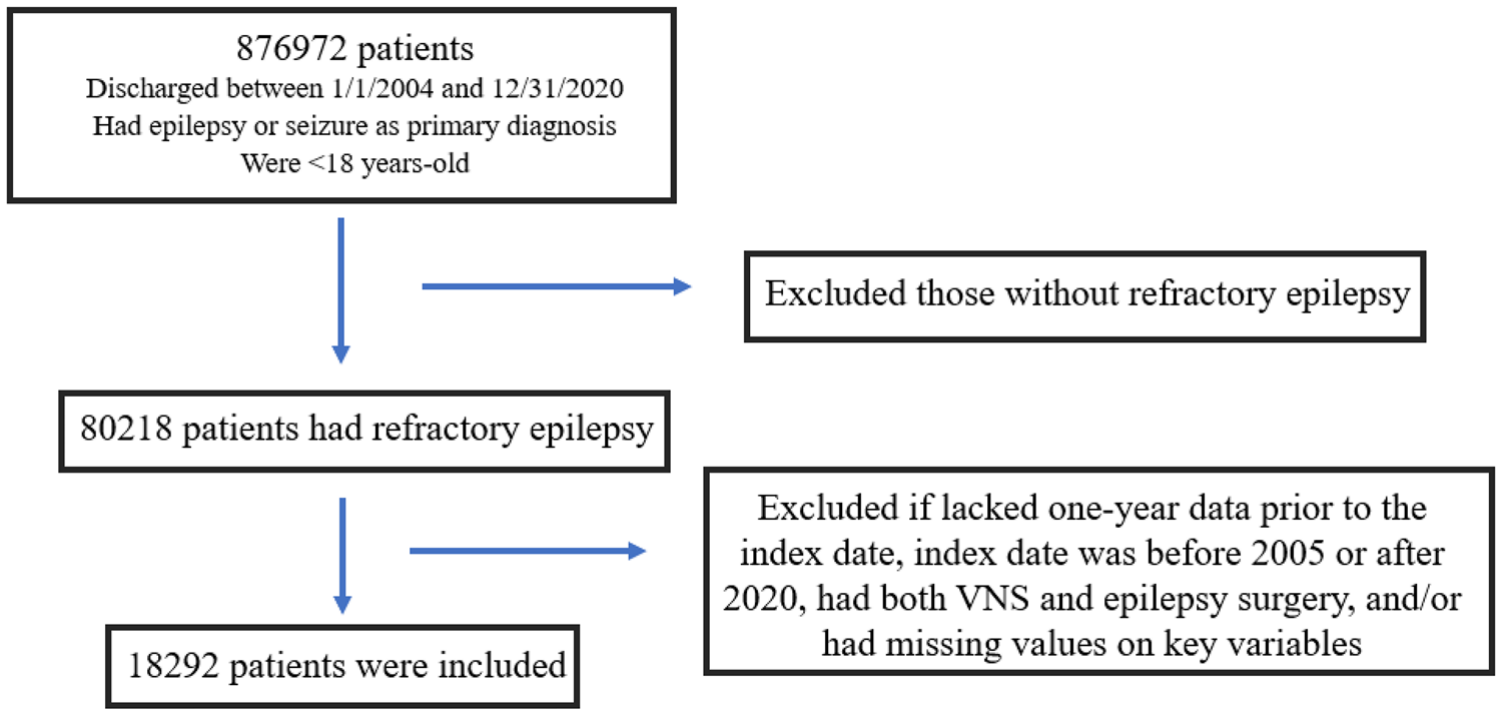
Flow diagram

A total of 18,292 patients were identified (Fig. 2); 10,240 treated with ASMs, 5019 treated with ASMs + VNS, and 3033 treated with ASMs + cranial epilepsy surgery.

Variations among the groups were examined. Sex was not found to significantly vary among groups. However, there was significant variation in age, US census region, race/ethnicity, patient type, presence of PCCCs, diagnosis, and insurance (*p* < 0.001). Those treated surgically, either with VNS or cranial epilepsy surgery, in addition to AEDs, were 2 years older than those medically treated (on at least 3 ASMs). This suggests an incremental time component for referral and/or surgical evaluation leading to surgery in those treated surgically compared to those with AEDs alone. There appeared to be a time lapse of 2 years to arrive at receiving surgery.

Patients treated with surgery in addition to ASMs, whether VNS or cranial surgery, were more often living in the South and Midwest (*p* = 0.001). Conversely, those medically treated were less likely to be living in the Midwest (25.46%). Those undergoing surgical treatment were more often identified as non-Hispanic white (51.78 vs. 66.47 and 63.60%, *p* < 0.001), have a focal/partial epilepsy diagnosis (874 vs. 10.86 vs. 30.10%, *p* < 0.001), and be privately insured (35.82 vs. 42.70 vs. 48.80%, *p* < 0.001). Those undergoing medical management were more often Hispanic (21.88 vs. 17.37 vs. 17.41, *p* < 0.001) and non-Hispanic black (18.43 vs. 9.64 vs. 10.12, *p* < 0.001) and had Medicaid insurance (58.85 vs. 48.44 vs. 40.29, *p* < 0.001). Differences were statistically significant in geographic region, race, and insurance status (Table 1).

**Table 1:** Preoperative factors associated with treatment type

**Characteristics**	**Total** ***N*** **= 18,292**		**AEDs only cohort** ***N *= 10,240**		**AEDs plus VNS cohort** ***N*** **= 5019**		**Surgery cohort** ***N*** **= 3033**		***p*** **value**
	*N*	%	*N*	%	*N*	%	*N*	%	
Age in yr									
< 4	3208	17.54	2304	22.50	463	9.22	441	14.54	< 0.001
4–11	9440	51.61	5318	51.93	2666	53.12	1456	48.01	
12–17	5644	30.86	2618	25.57	1890	37.66	1136	37.45	
Sex									
Male	9908	54.17	5516	53.87	2721	54.21	1671	55.09	0.490
Female	8384	45.83	4724	46.13	2298	45.79	1362	44.91	
Geographic region									
Midwest	4743	25.93	2607	25.46	1335	26.60	801	26.41	0.001
Northeast	2344	12.81	1414	13.81	561	11.18	369	12.17	
South	6973	38.12	3924	38.32	1922	38.29	1127	37.16	
West	4232	23.14	2295	22.41	1201	23.93	736	24.27	
Race and ethnicity									
Non-Hispanic white	10,567	57.77	5302	51.78	3336	66.47	1929	63.60	< 0.001
Non-Hispanic black	2678	14.64	1887	18.43	484	9.64	307	10.12	
Hispanic	3640	19.90	2240	21.88	872	17.37	528	17.41	
Other	1407	7.69	811	7.92	327	6.52	269	8.87	
Patient type at index date									
Inpatient	11,937	65.26	7796	76.13	1108	22.08	3033	100.00	< 0.001
Outpatient	5580	30.51	1679	7.47	3901	77.72	0		
ED	775	4.24	765	16.40	10	0.20	0		
Comorbidity with CCCs									
No	4311	23.57	3385	33.06	624	12.43	302	9.96	< 0.001
Yes	13,981	76.43	6855	66.94	4395	87.57	2731	90.04	
Primary diagnosis									
Focal/partial	2353	12.86	895	8.74	545	10.86	913	30.10	< 0.001
Generalized	1209	6.61	664	6.48	388	7.73	157	5.18	
Other	14,730	80.53	8681	84.78	4086	81.41	1963	64.72	
Insurance									
Medicaid	9372	51.24	5719	55.85	2431	48.44	1222	40.29	< 0.001
Private	7291	39.86	3668	35.82	2143	42.70	1480	48.80	
Other	1629	8.91	853	8.33	445	8.87	331	10.91	

*CCCs* Pediatric complex chronic conditions

Presence of comorbidity with CCC was more common in those treated surgically (66.94 vs. 87.57 vs. 90.04%, *p* < 0.001), whereas no presence of comorbidity with CCC was more common in those treated medically (33.06 vs. 12.43 vs. 9.96%, *p* < 0.001).

## Discussion

We conducted a retrospective cohort study examining the PHIS database for US pediatric patients with drug-resistant epilepsy (DRE) focusing on type of treatment received. We found that among those who underwent management with antiseizure medications only (ASMs), compared to those treated with medication and VNS (ASMs + VNS) or medication and cranial epilepsy surgery (ASMs + cranial surgery), were less likely to be living in the Midwest, identified as non-Hispanic white, have a focal/partial epilepsy diagnosis, and have private health insurance. Additionally, those treated surgically were, on average, 2 years older than those treated medically. These findings suggest that it takes up to 2 years for referrals and/or surgical evaluations leading to surgical treatment for DRE. The differences in the patient-level characteristics of groups receiving medical treatment versus surgical treatment for DRE suggest disparities in care associated with social determinants of health.

### Healthcare disparities in DRE in the USA

Healthcare in the USA is unique from healthcare in other countries. There is no universal system to assure access. Healthcare insurance is often private, associated with parental/caregiver employment. However, children are also eligible for government-sponsored insurance through Medicaid. Due to this, external conditions which impact the family and caregivers, such as employment, parental educational attainment, socioeconomic status, and environment, can impact healthcare access and engagement. For this reason, we explored the impact of geographical region by US census (Fig. 1) and insurance type on disparities in DRE care. We looked at this, in addition to demographics such as age, sex, race/ethnicity, and clinical factors such as epilepsy type and presence of comorbidities, to understand what role these and other determinants of health have in the context of the US healthcare system on epilepsy care in children.

**Fig. 3:**
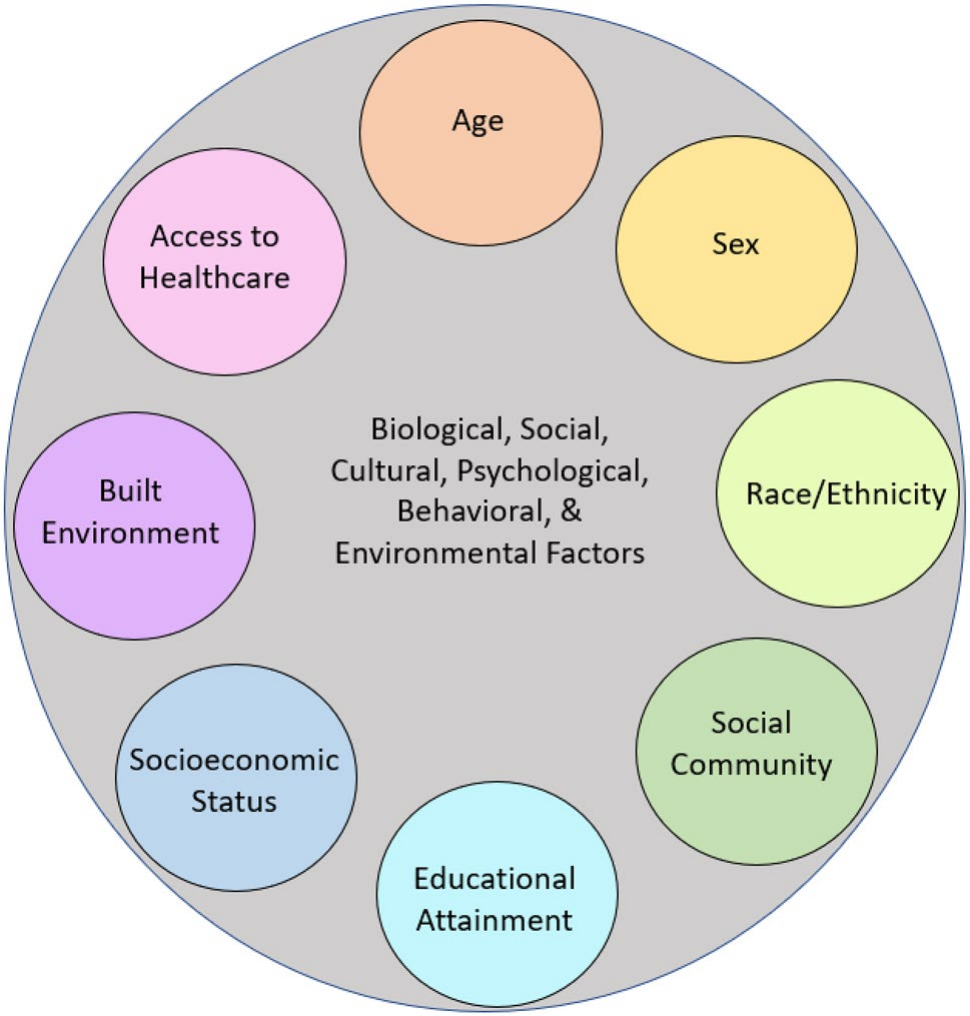
Social determinants of health affecting disparities in DRE

Social determinants of health (SDOH) are external conditions, including socioeconomic status, educational attainment, access to healthcare, neighborhood and built environment, and social community, which affect health, risk, and outcomes [12]. Factors such as race/ethnicity, age, and gender interplay with SDOHs like social/cultural, behavioral, biological, psychological, and environmental factors, contributing to complex relationships in engagement and participation in care, thereby leading to disparities in care and outcomes [5] (Fig. 3). To craft interventions aimed to resolve disparities, it is instrumental to understand what factors contribute to disparities and how societal, cultural, and personal factors contribute to healthcare [4, 6].

Our study identified age, race, geography, epilepsy type, insurance type, and presence of PCCCs to be associated with differences in DRE care. Those who were younger, of minority race/ethnicity, and had Medicaid insurance were more often treated medically than surgically. Those with PCCCs were more often treated surgically. Though patients with PCCCs have more comorbidities, they may already engaged in healthcare at tertiary care centers. Perhaps these tertiary centers are better equipped at expediting epilepsy work ups and surgical referrals. Additionally, perhaps the patients’ parents and caregivers are more engaged in the healthcare system and empowered to advocate more. We found that regional factors also influenced care, with those in the West and Northeast more commonly treated with epilepsy surgery compared to the Midwest and South. The West and Northeast tend to be more population dense than the Midwest and South in the USA. Perhaps this finding suggests that the higher the population density, the more common the incidence of DRE, the more streamlined the pathways. Alternatively, perhaps these variations by region may reflect findings attributable to different in rural communities compared to urban or suburban, or attributable to medically underserved communities.

These findings represent disparities in healthcare in DRE that may exist for a host of reasons, reflecting harder to quantify and qualify aspects of the physical, social, cultural, behavioral, biological, and psychological environments in which these patients live, impacting their access to and engagement with care [13, 14]. Many studies have aimed to understand the impact of these SDOHs in epilepsy and point to historical and structural etiology which are challenging to change without sweeping policy, advocacy, and engagement to promote equity; however, self-reflective identification and understanding, as well as competent medical education, and community-based practices are thought to present as key first steps [15].

### The impact of surgical epilepsy referrals in DRE

It is well-known that surgical treatment is the treatment modality of choice for epilepsy which is resistant to medical management alone [16]. However, despite this, delays exist. In 2001, it was postulated that the average patient lived with intractable epilepsy for 20 years before surgical epilepsy referral [17]. After recommendations and guidelines were enacted to encourage earlier referral for those with DRE, patients have been referred more commonly. However, the time to referral for evaluation varies widely, with some reporting an average of 38.3 weeks from diagnosis to referral, with time to treatment longer [18]. Referral times are considered worse for those living in rural regions with limited access to neurological care [19]. Furthermore, wait times are seen globally despite variations in healthcare systems and structures [20], underscoring that while this is a study of US patterns, similar problems may exist in other countries as well. While many studies point to system issues as etiologies for barriers in access and delays in referrals, provider and patient apprehension is another factor to consider [21]. Furthermore, social determinants of health additionally contribute to access to and engagement with care in these cases.

Coordination of epilepsy surgery is not simple. For these patients, extensive preoperative evaluation is necessary, involving comprehensive neurological assessment including multiple imaging modalities, video electroencephalography monitoring, and extensive coordination with a multidisciplinary team [22, 23]. Young patients may require sedation and anesthesia services for diagnostic studies such as neuroimaging. Often, pediatric DRE patients have comorbidities, reflected in pediatric complex chronic conditions (PCCCs) or other medical illnesses or syndromes which complicate their medical care coordination and delivery. Additionally, the impact of caring for a patient with DRE influences caregiver quality of life, contributing to caregiver burden and financial hardship which may impact engagement in healthcare [24]. However, it is known that DRE holds significant implications for the developing pediatric brain. Exposure to ongoing seizures may negatively impact health and neurocognitive outcomes. Seizure control is key. Thus, every effort should be made to mitigate impedances and enhance timeliness of referral to reduce time to treatment in children with DRE.

### Limitations

Limitations to this study are inherent in the use of administrative data: there may be errors in coding or documentation, surgical codes may not reliably reflect surgical innovations over the years of study (i.e., stereoelectroencephalography, magnetic resonance imaging-guided laser interstitial thermal therapy, endoscopic epilepsy surgery, or responsive neurostimulation), and granularity of clinical decision-making are not reflected. Furthermore, our statistical analysis can establish correlations, but causal relationships cannot be explained due to the limitations of observational data. Nevertheless, this study gives an important initial national perspective, describing the state of pediatric epilepsy care in terms of the characteristics of patients with DRE receiving medical treatment only, medications plus vagus nerve stimulation, and medications plus cranial epilepsy surgery.

In this study, we provide data for understanding patient-level characteristics related to treatment type. This study is the initial step in characterizing care of pediatric patients with drug-resistant epilepsy. Future directions include examining outcomes by specific treatment type and outcomes by patient-level and hospital-level characteristics.

## Conclusions

We studied a large administrative database examining variables associated with surgical epilepsy evaluation and management. We found significant variation in treatment associated with age, census region, race/ethnicity, patient type, presence of PCCCs, diagnosis, and insurance type. We believe that these disparities in care are related to access and social determinants of health, and we encourage focused outreach strategies to mitigate these disparities to broaden access and improve outcomes in children with DRE.

## Supplementary Information

Below is the link to the electronic supplementary material.

Supplementary file1 (DOCX 13.4 KB)

## Data Availability

Not applicable.
