# Supplementary material for: Disparities in pediatric drug-resistant epilepsy care
Source: Childs Nerv Syst. 2023 Feb 17;39(6):1611–7. doi: 10.1007/s00381-023-05854-y (PMC10227158; doi:10.1007/s00381-023-05854-y)
Supplement: Supplementary file 1 — Supplementary file1 (DOCX 13.4 KB) [file 381_2023_5854_MOESM1_ESM.docx]

Supplemental Table 1

Diagnosis Codes

| ICD-9-CM | 34501, 34511, 34541, 34551, 34561, 34571, 34581, 34591 |
| --- | --- |
| ICD-10-CM | G40011, G40019, G40111, G40119,G40211, G40219, G40311,G40319, G40A11,G40A19, G40B11, G40B19, G40411, G40419, G40803, G40804, G40813,G40814, G40823,G40824, G40911, G40919 |
